# Effect of Thermal Growth Oxide Composition and Morphology on Local Stresses in Thermal Barrier Coatings

**DOI:** 10.3390/ma15238442

**Published:** 2022-11-27

**Authors:** Kunying Ding, Tao Zhang, Zhe Wang, Jun Yu, Wansen Guo, Yifei Yang

**Affiliations:** 1 Tianjin Key Laboratory for Civil Aircraft Airworthiness and Maintenance, Civil Aviation University of China, Tianjin 300300, China; 2 Aircraft Maintenance and Engineering Corporation, Beijing 100621, China

**Keywords:** TGO dynamic evolution, thermal barrier coatings, microstructure, residual stress

## Abstract

The failure of thermal barrier coatings (TBCs) during operation depends mainly on the thermal mismatch between the ceramic top coat (TC) and the metal bond coat (BC). The thermal mismatch at the interface is influenced by the dynamic changes in the composition and morphology of the thermally grown oxide (TGO) between TC and BC during thermal cycling. This work focuses on the establishment of a TGO dynamic growth model, which considers the changes in TGO composition and morphology for investigating the effect of dynamic growth of TGO on local mismatch stresses during thermal cycling. The results show that the sharp locations at the TGO/BC interface are more prone to high tensile stresses during thermal cycling due to the uneven growth behavior of TGO, leading to crack initiation. The valley region of the interface is in a state of compressive stress σ_xx_ during the early stages of thermal exposure. The peak region preferentially forms a concentration of tensile stress σ_yy_. Once large-scale “layer” (Ni, Co)Al_2_O_4_-based spinel-like mixed oxides(MO) growth occurs in TGO, the stress σ_xx_ changes from compressive stress to tensile stress in the valley region, eventually forming high tensile stress (Max: +158 MPa). The maximum tensile stress σ_yy_ in the peak region is increased to 256 MPa, which is more than two times larger than the early period of thermal exposure. As a result, the dramatic changes in local stresses seriously affect the time and location of microcracks.

## 1. Introduction

Thermal barrier coatings (TBCs) have been widely used on aero-engine hot-section components to significantly improve the resistance in high temperature, oxidation, and corrosion [1]. A conventional TBC system usually consists of yttria stabilized zirconia (YSZ) as a top coat (TC), an MCrAlY (M = Ni and/or Co) oxidation-resistant metallic bond coat (BC) and, for example, a Ni-based superalloy as a substrate [2,3,4,5]. During operation, a layer of thermally grown oxide (TGO) is formed between TC and BC of the TBCs. The interfacial mismatch stress in TBCs rises further due to the thickening of the TGO layer, eventually causing TC spalling or delamination [6]. In recent years, it has been noticed that the TC/BC interface is usually uneven in atmospheric plasma spray (APS) systems of TBCs. The variability of elemental diffusion at local locations was affected by this interfacial morphology, resulting in the growth of TGO in an irregular form [7]. The complex local stresses within the coating were affected by the combination of growth behavior and interfacial thermal mismatch [8], causing preferential crack nucleation at certain locations in the interfacial region [9].

Scholars have focused their research attention on the impact on the TGO growth process. Che et al. [10] demonstrated that the growth rate of TGO was higher in the peak interface than that in the valley region under an Isothermal test at 1050 °C. Busso et al. [11] revealed that the maximum tensile stress in the peak region of the TGO/BC interface (+500 MPa) was much higher than that of other regions when the amplitude (b)/half wavelength (a) of the interface morphology = 1, having the trend to produce a subcritical crack. Kyaw et al. [12] and Song et al. [13] further found a larger concentration of tensile stresses generated in the near-peak region during the TGO uneven growth process. Kim et al. [14] observed that the maximum stress region gradually shifted from the peak position to the valley situation in a 3D damage model under the thermal cycling process, and this situation matched well with the actual test. Zhang et al. [15] observed that the spinel with a far higher growth rate than Al_2_O_3_ has a more critical effect on the coating failure through TGO growth behavior in APS-TBCs. Xie et al. [16] analyzed the effect of mixed oxide (MO) growth on the stress evolution and found that the MO in the peak region was responsible for serious interfacial tensile stresses, which accelerate the crack propagation in the interface.

The above studies generally concluded that the TGO composition and morphology influenced the stress distribution and magnitude of TBCs. However, the effect of thermal mismatch stress accumulation during thermal cycling cannot be ignored. Meanwhile, the important influence that considering the evolutionary process of TGO composition, morphology and stress accumulation played in the dynamic development of local stress in TBCs remained unclear until now. TGO growth behavior based on oxidation kinetics is necessary for the investigation of local stresses in TBCs. Unfortunately, the existing models do not adequately consider the effects of TGO composition and morphology on the local stress distribution and development of TBCs under stress accumulation.

In the current work, the regional growth model of staged oxides (Al_2_O_3_ and MO) is proposed to investigate the effect of TGO composition and morphological evolution on the interfacial mismatch stress of the coating under thermal cycling. Considering creep and thermal stress accumulation, a novel stress analysis model for TBCs is developed and validated by comparing the results with photoluminescence piezo-spectroscopy (PLPS) detection. The present model not only refines the effect of the local growth behavior of TGO during thermal cycling on the stress magnitude, distribution, and evolution of TBCs but also predicts the location and time of microcrack appearance. This work provides new support for the problem of life prediction of surface coatings on hot-section components.

## 2. Experimental Process and Analysis Model

### 2.1. Preparation of Thermal Barrier Coating and Thermal Cycling Test

**Figure 1:**
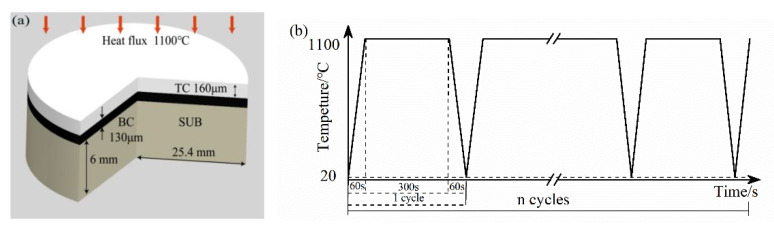
Coating structure and thermal cycling process: (**a**) 8YSZ coating structure and (**b**) cyclic thermal loading history.

Hastelloy-X alloy was chosen as the substrate material. The substrate was disc-shaped, and the size was φ25.4 mm × 6 mm. Before spraying, the substrate specimens were first cleaned with acetone, and then the sample surface was sandblasted with white corundum grit (The equipment was 8070P-B type sand blasting machine produced by Jichuan Machinery Technology Company). Commercial MCrAlY powder, PWA1348-2, was used as BC and was deposited with APS to obtain a nominal thickness of 130 μm. The TC was made from 8 wt.% YSZ and deposited using APS to obtain a nominal thickness of 160 μm. The used equipment was a Praxair 3710 plasma arc spraying system made by Praxair TAFA and 2400M six-axis manipulator made by ABB. All experiments was performed at room temperature (20 °C) under laboratory conditions. The coating structure is shown in Figure 1a, and the technological parameters during spraying are listed in Table 1.

Thermal cycling tests were performed on TBCs using the thermal process in Figure 1b (1100 °C for 5 min, air cooling to room temperature 20 °C) and analyzed for stresses and microcracks.

### 2.2. Analysis of Thermal Barrier Coating Morphology

The cross-sectional morphology of the coatings was observed and analyzed using a TESCAN MIRA LMS-type scanning electron microscope (SEM). Three typical areas were selected from samples with different numbers of thermal cycles. Image-Pro Plus 6.0 image analysis software was used to measure the TGO thickness in the selected areas. The following equation calculates the equivalent thickness of the TGO layer (Equation (1)):

δ_TGO_ = ∑S/∑L (1)

where δ_TGO_ is the equivalent thickness of TGO in the coating/μm; ∑S is the area of TGO/μm^2^; ∑L is the average value of TC/TGO and TGO/BC interface length/μm.

**Figure 2:**
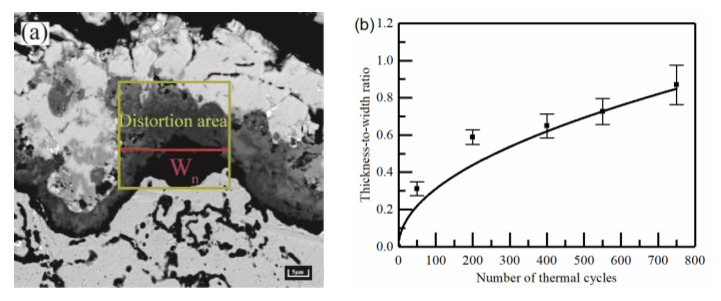
Thickness to width ratio of peak: (**a**) Thickness-to-width ratio diagram and (**b**) Results of thickness to width ratio under thermal cycling.

The samples were sprayed with the same technological parameters for the process. The SEM images of 10 representative interfacial oscillation cycles were selected from the sample images with the different numbers of thermal cycles. The thickness-to-width ratio of the peak ε_n_ was introduced by the position of the aberrations within the cycle (as shown in the yellow rectangular area in Figure 2). The peak region is determined with Equation (2) to provide the fundamental basis for the geometric modelling.

ε_n_ = L_n_/W_n_ (2)

where L_n_ is the equivalent size for the different number of thermal cycles in each oscillation cycle of the typical region/μm, and W_n_ is the width of the aberrant region/μm, as shown in the red line in Figure 2a. The valley region was not clearly delineated by features, then the same width range as the peak region was used. Figure 2b shows the thickness-to-width ratio of the peak under different thermal cycle times.

**Figure 3:**
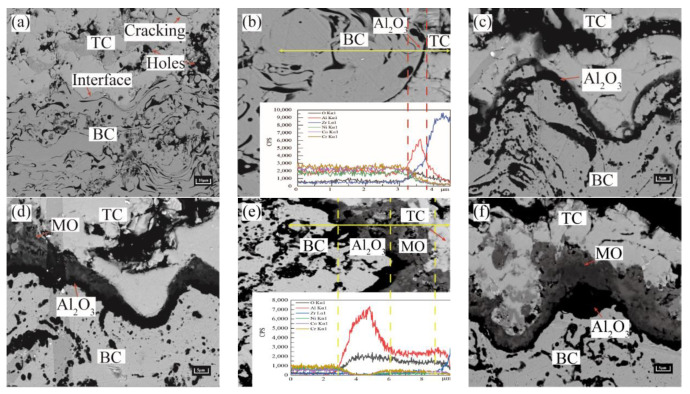
SEM images of cross-section of 8YSZ coating: (**a**) TBCs in the sprayed state, (**b**) 50 cycles, (**c**) 200 cycles, (**d**) 400 cycles, (**e**) 550 cycles, and (**f**) 750 cycles.

**Figure 4:**
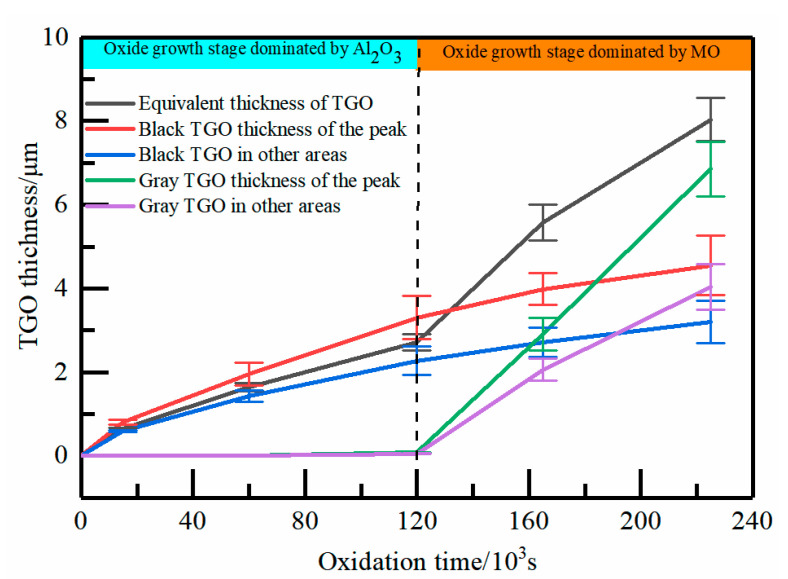
Statistical results of TGO thickness in different areas of the TC/BC interface.

The growth of TGO layers was analyzed according to the cross-sectional morphology of TBCs with different numbers of thermal cycles (Figure 3). It can be observed from Figure 3a that a large number of holes and microcracks emerged in the TC layer in the original state. The TC/BC interface was uneven and in the shape of a cosine function. There is no oxide at the interface between the TC and BC layers at this state. After 50 thermal cycles (Figure 3b), the selective oxidation of Aluminum occurs at the TC/BC interface due to the higher Gibbs free energy (Al^3+^ > Cr^3+^ > Co^2+^ > Ni^2+^), preferentially forming α-Al_2_O_3_ based TGO, as shown in Table 2. After 200 thermal cycles (Figure 3c), an unbroke dense black TGO layer dominated by α-Al_2_O_3_ was formed at the TC/BC interface. After 400 thermal cycles (Figure 3d), the thickening of the Al_2_O_3_ layer extended longitudinally, which appeared as a local thickness unevenness and produced a trace of gray mixed oxide (MO). After 550 thermal cycles (Figure 3e), the gray MO layer grew rapidly, especially in the peak region, leading to a further increase in the fluctuation at the interface. The excessive boundary between MO and Al_2_O_3_ can be clearly observed in the spectrum. After 750 thermal cycles (Figure 3f), the difference in thickness between regions was further increased due to uneven growth. The statistical results of the TGO thickness under different regions are shown in Figure 4.

As shown in Figure 4, the TGO thickness increased slowly in each region during the pre-oxidation stage. The thickening rate of TGO in the peak region was slightly higher than that in other regions. MO began to appear with the deepening of the oxidation process. It is clearly seen from the figure that the thickness of the MO layer increased rapidly on the entire boundary, especially in the peak region, which was about 6.860 μm. The thickening of the Al_2_O_3_ layer became extremely slow, with a peak region of about 4.551 μm.

### 2.3. Calculation of Oxidation Kinetics

To characterize the TGO growth further precisely, this work introduced the regional growth rates of Al_2_O_3_ and MO under different oxidation processes by thickness-to-width ratios. The growth rate provided data to support the construction of the dynamic growth model of TGO.

Equation (3) can be derived from Wagner’s high-temperature oxidation theory [18]:

(3) $$h={\left[2D_{V}C_{1}\int_{0}^{t}\exp\left(\frac{\sigma_{y}\Delta \Omega }{kT}\right)dt\right]}^{\frac{1}{2}}$$

where h is the local thickness of the TGO layer, *D*_v_ is the diffusion coefficient, *C*_1_ is the oxygen vacancy concentration on the outer surface of the oxide film at the interface between the oxide film and the metal, *k* and *T* are Boltzmann constant, and thermodynamic temperature, respectively, σ_y_ is the internal stress of the oxide film (the oxide growth rate is mainly related to the *y*-direction stress), and ∆Ω is the activation volume during the oxidation process.

The TGO thickness was positively correlated with the *y*-directional stress, meaning that the increase of *y*-directional stress promoted the rise of tensile stress, while the vacancy concentration inside the TGO enhanced the diffusion ability of elements at the interface in Equation (3). The strength of the element diffusion ability directly determined the TGO growth rate. The “wave-like” morphology of the APS technology accelerated the growth of TGO at high tensile stress locations, and the uneven growth further increased the stress in the coating. In order to highlight the effect of TGO growth evolution on coating stresses in the peak region, the model was divided into two parts in the definition of the TGO growth region: the peak region and the other regions.

The Wagner parabolic growth law (Equation (4)) was used to characterize the local growth rate of TGO at 1100 °C.

*h*_TGO_ = *K*·*t*^0.5^ (4)

where *h*_TGO_ is the local thickness of the TGO layer and t is the time (insulation phase only). To accurately analyze the effect of TGO composition and morphological evolution on stress, the TGO growth process in each region was simplified into two stages (Figure 4): The first stage was the initiation and “layer” growth of Al_2_O_3_. The second stage was the rapid growth of MO. The growth rates of TGO regions under different oxidation processes are shown in Table 3. The physical geometry model was constructed separately based on the growth rates of oxide regions under different oxidation stages.

### 2.4. Model Size, Meshing, and Boundary Conditions

**Figure 5:**
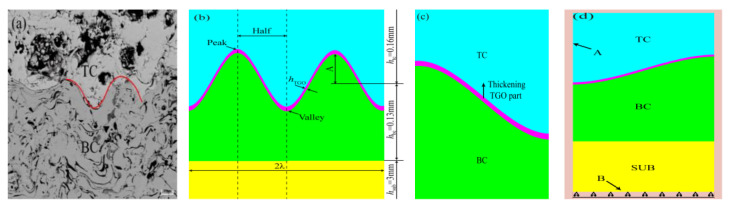
Model of thermal barrier coating: (**a**) the actual appearance of thermal barrier coating (**b**) two-dimensional model of the TBC system (**c**) TGO growth direction (**d**) temperature and displacement boundary conditions.

To establish the model of TC/BC interface morphology and the effect of TGO growth on stress under thermal cycling, ANSYS 2020 R1 software was used to analyze it. The ideal model of the 2D cosine curve was used to construct the TC/BC interface morphology in this work based on the real morphology of TBCs under APS technology (Figure 5a). The amplitude (A) was 10 μm, and the wavelength(λ) was 100 μm. The TBCs model in the initial state consists of three layers: SUB, BC and, TC, with thicknesses of 3 mm, 0.13 mm, and 0.16 mm, respectively (Figure 5b).

The FE model was divided by quadrilateral meshing. The locations near the TC/BC boundary were divided using a more dense equal-sized surface mesh (the cell type was Plane77. The mesh cell size was 0.5 μm and each cell contained 4 nodes). This can better control the TGO growth process and reserve space for TGO growth along the *y*-direction, as shown by the arrow in Figure 5c.

The temperature load was applied to the outer boundary of the model according to the thermal cycling process in Figure 1b, as shown by arrow A in Figure 5d. Each thermal cycle lasted 420 s, and a total of 20 cycles were analyzed to simulate 750 thermal cycles under actual conditions. Constraining the freedom in the *y*-direction at the lower edge of the model (setting the frictionless support at y = 0) provided a more realistic description of the stress-strain variation in the TBCs under laboratory conditions, as shown by arrow B in Figure 5d.

### 2.5. Material Parameters

To conveniently investigate the stress evolution and distribution in TBCs, each layer was considered as a homogeneous, isotropic linear elastic material during the simulation. The layers of material are small deformations with 2D plane strain. The specific material parameters of each layer can be seen in Table 4 [19]. The coating operated in the temperature range of 20 °C to 1100 °C and crept in high temperature environments. The stress on the coating can be significantly affected by the increasing number of thermal cycles. Therefore, the creep properties of the material were considered in the calculation process. Norton’s power law creep is shown in Equation (5).

*ε*_cr_ = B/σ^n^ (5)

where *ε*_cr_ is the strain rate/s^−1^, B is the pre-factor /s^−1^MPa^-n^, σ is the stress/MPa, and n is the power-law creep exponent. The creep parameters are shown in Table 5 [20].

### 2.6. TGO Growth Model

**Figure 6:**
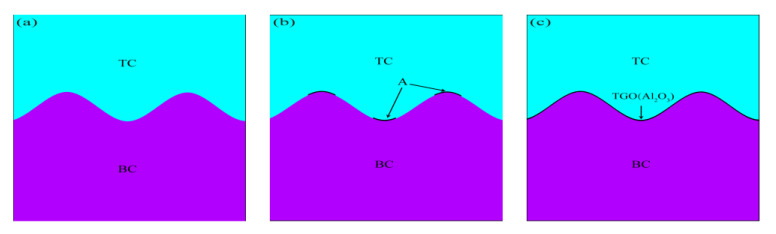

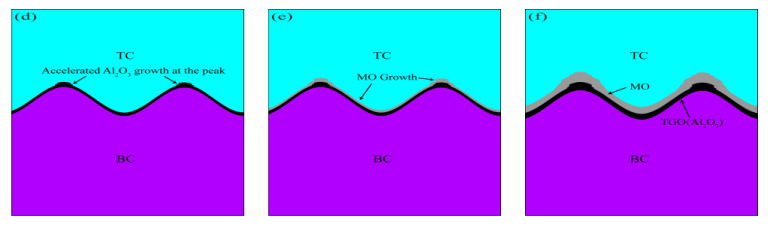
Dynamic growth model of TGO: (**a**) C = 0, (**b**) C = 3, (**c**) C = 5, (**d**) C =10, (**e**) C = 12, and (**f**) C = 20.

The growth process of TGO was characterized according to the growth rate of regional oxides under different stages. A compiled program for dynamic growth of TGO by growth parameters was developed, and ANSYS was used to analyze the stress of TBCs during thermal cycling. The growth morphology of TGO in each stage is shown in Figure 6. In order to simplify the number of simulations and ensure the reliability of the stresses, the test process of 750 thermal cycles was transformed into 20 thermal cycles to simulate the overall TGO growth process (C is the number of thermal cycles). The process was refined into four main stages: (1) C = 1–2 thermal cycles correspond to the morphology of TBCs without TGO (Figure 6a). (2) C = 3–4 thermal cycles correspond to the longitudinal slow growth phase of Al_2_O_3_ at the TC/BC interface. Al_2_O_3_ starts to emerge from the peak to the valley at C = 3, as shown by arrow A in Figure 6b. (3) C = 5–10 cycles correspond to the slow growth stage of the Al_2_O_3_ longitudinal “layer”. An even and dense layer of Al_2_O_3_ had formed at the TC/BC interface at C = 5, as shown in Figure 6c. The uneven growth of the TGO layer at the TC/BC interface was clearly observed at C = 10, as shown in Figure 6d. (4) C = 11–20 thermal cycles correspond to the longitudinal rapid growth phase of MO. The MO grew explosively at C = 12, as shown in Figure 6e. The MO in the peak region still kept growing rapidly, and eventually the maximum thickness of TGO in the peak region reached 12 μm at C = 20 (Figure 6f).

## 3. Results and Analysis

### 3.1. Residual Stress in the TGO Layer

**Figure 7:**
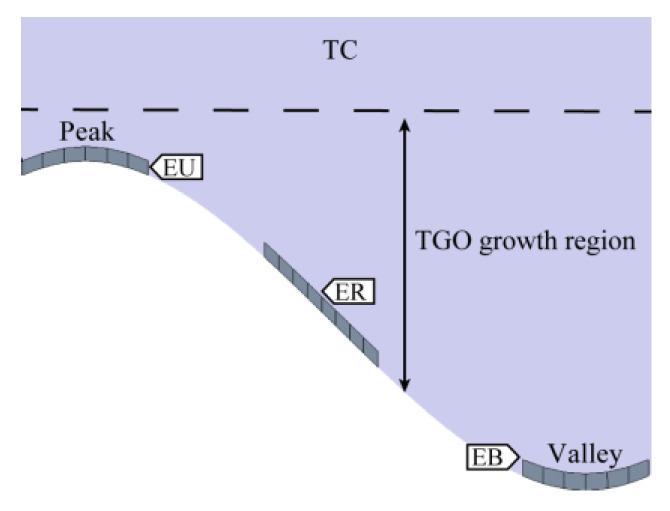
Typical regions in the TGO.

Regional differences in the oxidation process can cause complications in oxide growth. In this study, three representative regions within the TGO, namely, the peak region (EU), the valley region (EB), and the ramp region (ER), were analyzed separately. Figure 7 shows the schematic diagram of typical regions within the TGO.

**Figure 8:**
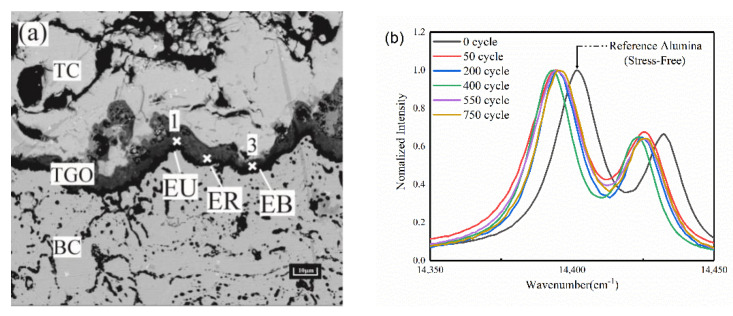
Test area and corresponding energy spectrum using PLPS: (**a**) Test area diagram and (**b**) One group of energy spectrum test graphs.

The TGO layers under different thermal cycles were tested using a Raman microscope (Horiba LabRAM HR, Kyoto, Japan) with an excitation of 532 nm. The TGO stress was characterized by tracking the frequency shift of R2 of Cr^3+^ in α-Al_2_O_3_ [21]. Figure 8a shows a schematic of the PLPS test area, and Figure 8b shows the tested PLPS spectra of the TGO [22].

The collected spectral curves were fitted with the Gaussian Lorentz function by Labspec 6 spectral analysis software, and the fitting accuracy was about 0.5 cm^−1^. The TGO can be considered under biaxial stress due to its flat structure. Lipkin and Clarke et al. [23,24] discussed the stress tensor of TGO in more detail. The residual stress σ in the TGO can be calculated by Equation (6) [25].

∆υ = 5.07σ(6)

where Δυ is the frequency shift of R2 fluorescence. All the Raman measurements were carried out at room temperature(20 °C).

**Figure 9:**
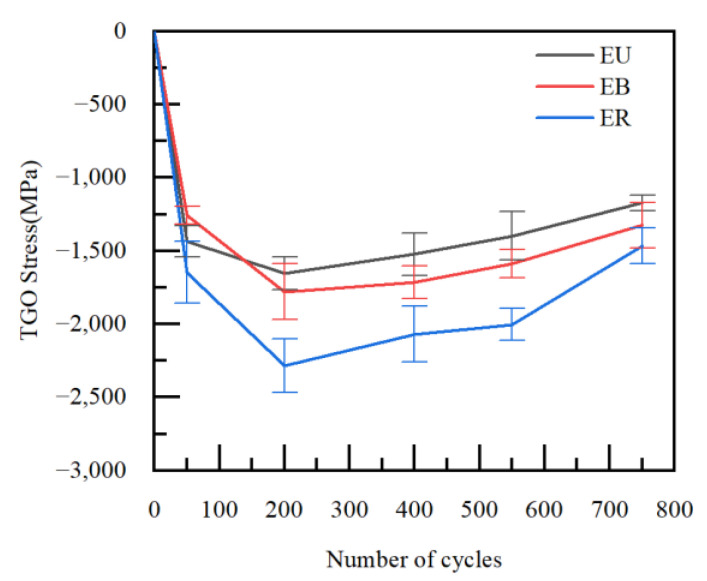
Residual stresses in the TGO under thermal cycling.

**Figure 10:**
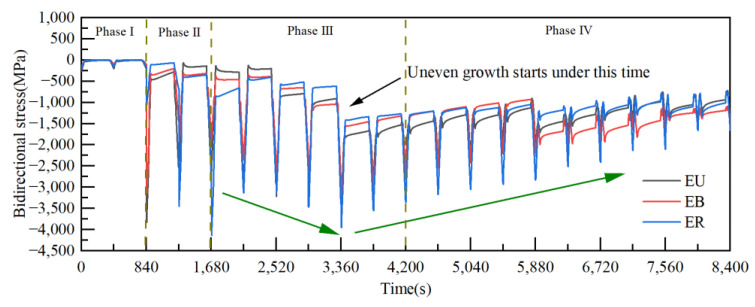
Regional biaxial stress diagram in the TGO under thermal cycling.

The stresses in each region in the TGO were calculated for different numbers of thermal cycles. Figure 9 shows the magnitude of residual stresses in the TGO region after 50, 200, 400, 550 and 750 thermal cycles of the TBCs. Figure 10 shows the regional biaxial stress diagram in the TGO during thermal cycling.

The test results from Figure 9 showed that the stress states of TGO under the cooling stage were compressive stresses. The residual stresses in the three regions were relatively close to each other after 50 thermal cycles. A dense layer of TGO was formed at 200 thermal cycles, and the compressive stress reached the maximum at this time, then began to decrease slowly. The compressive stress reached the minimum after 750 thermal cycles. The test results were consistent with the results of Xu [26] and with the biaxial stress results (The biaxial stress is the sum of the principal stress vectors in both directions) for each region at room temperature in the FE model, as shown in Figure 10. The result can further verify the accuracy of the constructed model and stress evolution based on oxidation kinetics in this work, and also provide the basis for analyzing the dangerous loading in the coating.

### 3.2. Effect of Thermal Cycling Process on Stress Distribution in TGO Layer

The failure of coatings is mostly defined by crack initiation and the crack generation drived by two stresses σ_xx_ and σ_yy_ in the TGO layer [27,28]. Therefore, the study first examined the evolution of stresses σ_xx_ and σ_yy_ in the TGO layer during thermal cycling.

**Figure 11:**
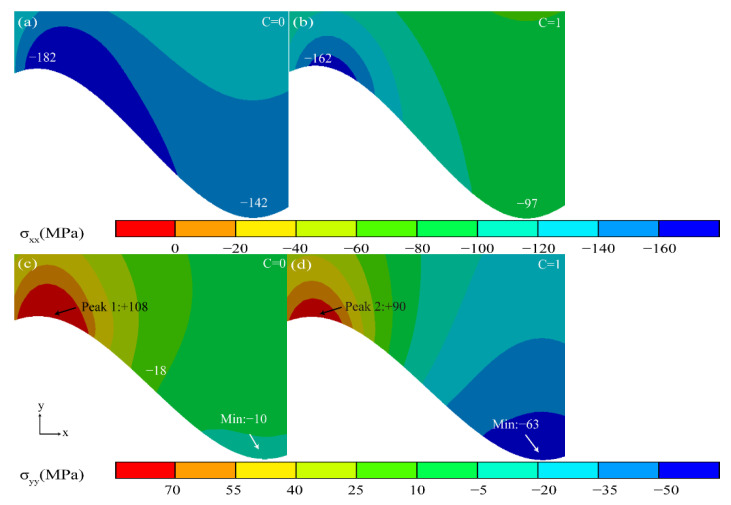
Stress diagram of the coating in the ungrown state of TGO.

Figure 11 shows the stress diagram of the TGO in the ungrown state. In the *X* direction, the TC layer in the original state was in a compressive stress state. The stress in the coating was released and the compressive stress near the TC/BC interface started to decrease as the thermal cycle started (Figure 11b).

In the *Y* direction, the tensile stress in the peak region (Peak 1) in the original state was the largest at +108 MPa. The stress gradually changes from tensile stress to compressive stress from the peak region to the valley region. The tensile stress in the peak area (Peak 2) was gradually released as the thermal cycle started.

**Figure 12:**
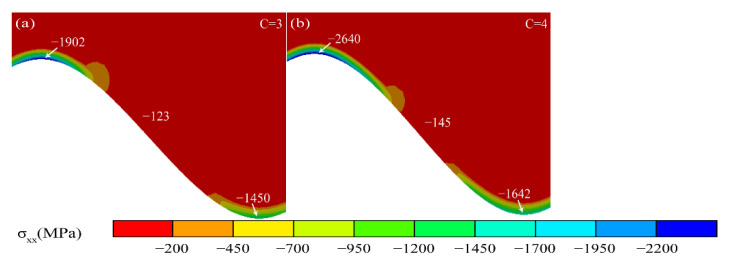

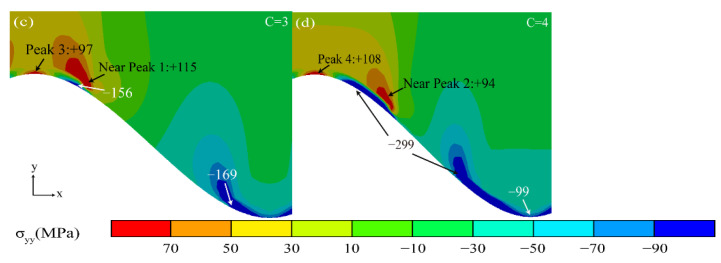
Stress diagram of TGO layer under Al_2_O_3_ sprouting process.

Figure 12 shows the stress diagram under the Al_2_O_3_ initiation process. In the *X* direction, high-pressure stress concentrations appeared in the peak and valley regions due to the Al_2_O_3_ initiation. The compressive stresses and their areas increased as the TGO grew.

In the *Y* direction, the stress distribution near the interface becomes very heterogeneous during the Al_2_O_3_ initiation process. The tensile stress in the peak region (Peak 3, 4) of the TGO layer kept rising. The high tensile stress region also appeared in the near peak region (Near Peak 1, 2) near the TC/TGO interface and moved with the TC/TGO interface.

**Figure 13:**
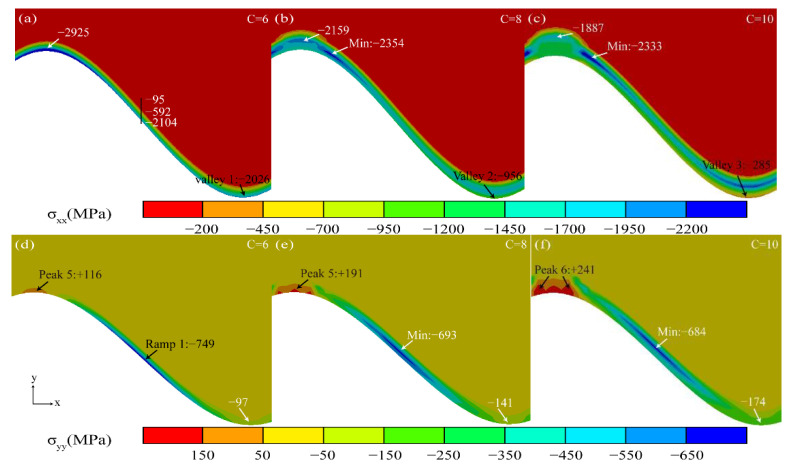
Stress diagram of TGO layers under the longitudinal “layer” growth phase of Al_2_O_3_.

Figure 13 shows the stress diagram under the longitudinal “layer” growth phase of Al_2_O_3_. With the longitudinal growth of Al_2_O_3_, the compressive stress in the valley region (Valley 1, 2, 3) near the TGO/BC interface gradually decreased in the *X* direction.

In the *Y* direction, the maximum tensile stress remained in the peak region near the TGO/BC interface (Peak 5, 6, 7). The relatively even compressive stress distribution in the ramp region (Ramp 1) can protect the thermal barrier coating more effectively [29].

**Figure 14:**
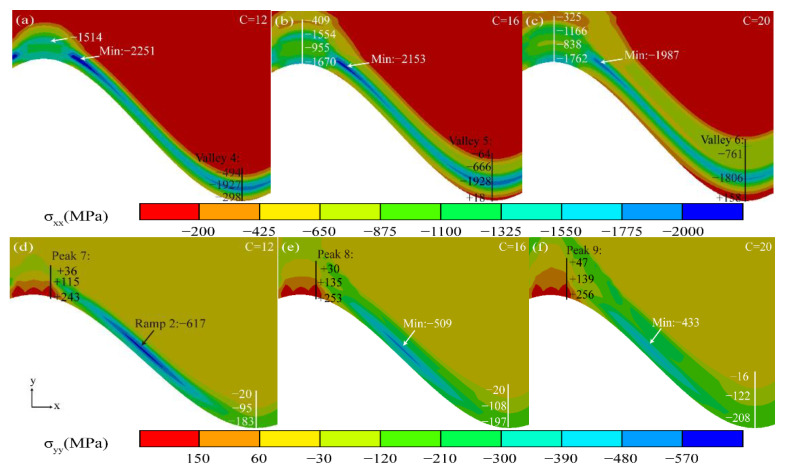
Stress diagram of the TGO layer under rapid growth of MO.

Figure 14 shows the stress diagram for the rapid growth phase of MO. The transition from compressive stress (−298 MPa) to tensile stress (+158 MPa) occurred in the valley region near the TGO/BC interface (Valley 4 to 6), eventually maintaining a high tensile stress concentration zone in the *X* direction.

The maximum tensile stress in the peak region near the TGO/BC interface (Peak 7, 8, 9) increased from +243 MPa to +291 MPa in the *Y* direction. The “compressive stress zone” remained in the ramp area of the Al_2_O_3_ layer (Ramp 2), but the stress decreased sharply, causing the protection of the coating to also decrease.

The stress distribution of the above thermal cycles observed that the maximum tensile stress σ_yy_ occurred in the near-peak region close to the TC/TGO interface (Near Peak 1–2) during the initial few thermal cycles (3–4 cycles) of the Al_2_O_3_ initiation. Maximum tensile stress σ_yy_ changes the position to the peak region near the TGO/BC interface (Peak 1–9) with the increasing number of thermal cycles (5–20 cycles). The stress σ_xx_ changed from compressive to tensile stresses in the valley region near the TGO/BC interface (Valley 1–6) during the rapid growth of MO. It can be inferred that the continuously growing tensile stresses in both regions tended to be the driving force for crack initiation.

### 3.3. Stress Evolution Law in Typical Regions

**Figure 15:**
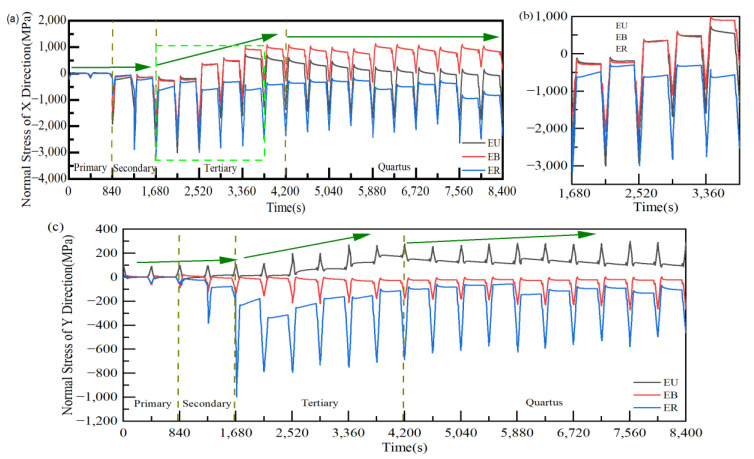
Effect of thermal cycling cycles on the stress evolution in typical regions of the TGO layer. (**a**) Stress evolution in the x-direction (**b**) Stress transition in X direction and (**c**) Stress evolution in the x-direction.

For a clearer analysis of the correlation between the TGO growth behavior and the stress evolution under different regions, the stresses σ_xx_ and σ_yy_ in typical regions were investigated separately in this work (Figure 15). The stress changes in the three regions were similar and relatively small in the TGO ungrown state in Figure 15a. The maximum compressive stress under all three regions during Al_2_O_3_ initiation tended to increase, especially in EU. The maximum compressive stress in the three regions slowly decreased during the slow growth of the “layer” of Al_2_O_3_. With the appearance of the difference in oxide growth rate in the TGO region, the stresses in the EU and EB regions gradually changed from compressive stresses to tensile stresses during the holding period, and the EB tensile stress growth rate was larger in Figure 15b. EB was increasingly affected by tensile stress from the beginning of the explosive growth of MO. Thus, it can be shown that the tensile stress concentration region appeared in the valley region since the beginning of the oxidation layer uneven growth compared to other regions. The tensile stress also increased gradually with the growth of MO, which is more prone to crack initiation and propagation under stress σ_xx_.

As shown in Figure 15c, it can be seen that the maximum tensile stress of EU was around +100 MPa at no TGO. The tensile stress of EU increases slowly with Al_2_O_3_ initiation, from 97 MPa to 108 MPa. The ER generated enormous compressive stress due to the extrusion of the initiated TGO. The maximum tensile stress in the EU increased with the TGO thickness at the beginning of the “layer” growth of Al_2_O_3_. With the rapid growth of MO, the maximum tensile stress of the EU kept growing. It can be concluded that the oxide growth had a significant effect on the growth of tensile stress in the peak region within the TGO, especially at the uneven growth of Al_2_O_3_, which was more prone to microcracking under stress σ_yy_.

**Figure 16:**
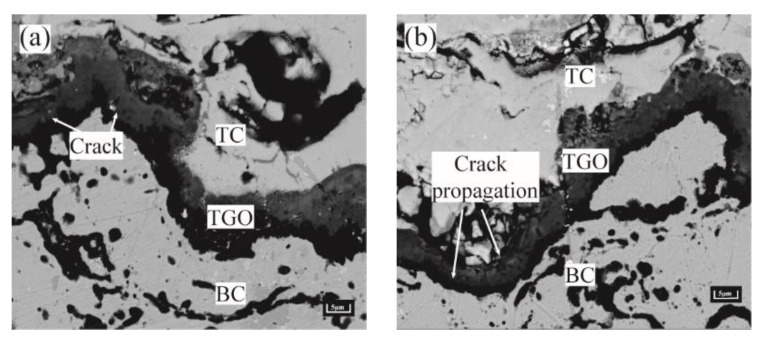
Crack initiation and propagation near BC/TGO interface. (**a**) Cracking in the peak area and (**b**) Cracking in the valley region.

The regional stress evolution law above speculated that the peak region of the TGO layer was more prone to microcracking at Al_2_O_3_ uneven thickening. The microcracks in the valley region were prone to appear under the MO rapid growth phase. The presence of crack initiation and propagation in the peak and valley regions can be seen in the samples after long thermal cycling (Figure 16). The phenomenon was consistent with the conclusion reached by Karadge et al. [30].

The average crack length was calculated for each group of samples using five typical SEM images of successive cracks selected at TGO/BC interface. As shown in Equation (7).

L = L_TC_/N (7)

where L is the average crack length, L_TC_ is the sum of all crack lengths, and N is the number of cracks near the interface. To summarize the relationship between various factors according to the number of thermal cycles, TGO growth behavior, stress and crack initiation and propagation are shown in Table 6. The effect of thermal cycling on the evolution of stresses and cracks was reflected in a quantitative way. The stresses in both directions were essentially unchanged at the early stage of the thermal cycle (C ≤ 4) in the table. The stress growth rate in both directions was larger in the middle of the thermal cycle (4 < C ≤ 10). The stress growth rates in both directions were larger, however the maximum failure strength was not reached in the middle of the thermal cycle (4 < C ≤ 10). The crack propagation rate was slower in the peak and valley regions. The growth rate of tensile stress was slowed down by the appearance of spinel. Tensile stresses exceed the maximum failure strength at the late stage of thermal cycling (C > 10). The rate of interfacial crack propagation was significantly higher. The presence of the valley region produced σ_xx_ above +1500 MPa and σ_yy_ above +250 MPa in the peak region eventuallyaccording to Wang et al. [31]. This was consistent with the numerical results in the current model. This indicated that there was a strong correlation between the elevation of tensile stress, crack propagation rate, TGO composition and morphology.

## 4. Conclusions

A TGO dynamic growth model was developed based on the regional oxide growth rates under different oxidation processes. The effect of the TGO growth process on the local stress field of the coating during thermal cycling was investigated. In addition, the stress data obtained by PLPS were verified. The main conclusions that can be drawn from this study are as follows.

(1) As observed from the TBCs cross-sectional morphology and the growth behavior of regional TGO, it was known that the oxide growth was the fastest in the peak region of the TC/BC interface, which was 1.4–1.8 times higher than the other regions, especially in the MO growth phase. The phenomenon indicated that the TGO growth behavior was strongly correlated with the local morphology and oxide composition of the interface, providing data to support the development of an accurate FE model.
(2) According to the PLPS test results and the TGO biaxial stress evolution law, it can be seen that the TGO local residual stress trend can be accurately characterized using the biaxial stress results. If the TGO residual compressive stress decreases significantly in the stress test, a non-destructive examination of the coating is required.
(3) The valley region near the TGO/BC interface was in the state of compressive stress σ_xx_, and the maximum tensile stress σ_yy_ (+116 MPa) existed in the peak region in the early stage of thermal exposure. The stress changes in the peak and valley regions were accelerated by the uneven thickening of the Al_2_O_3_ layer. Once the rapid longitudinal growth of the large-scale MO layer (h_MO_ ≥ h_Al2O3_) occurred, the stress σ_xx_ in the valley region started to change from compressive stress to tensile stress and eventually formed a tensile stress concentration zone (Max: +158 MPa). The tensile stress σ_yy_ in the peak region was also increased to 256 MPa, which was more than two times larger than the early period of thermal exposure. Therefore, the TGO composition and morphology were the main factors to generate severe tensile stresses.
(4) The high tensile stress concentration near the interface severely affects the location and propagation of the cracks. The maximum tensile stress σ_yy_ was in the peak region during the uneven thickening of the Al_2_O_3_ layer. Cracks may be preferentially nucleated in the region under this stage. In contrast, the tensile stress σ_xx_ was concentrated in the valley region at the appearance of large scale “layer” MO growth, leading to the appearance of microcracks. The persistent high tensile stress (σ_xx_) eventually resulted in larger average crack length in the valley region (L_V_:39.37 μm > L_P_:34.31 μm).

## Figures and Tables

**Table 1:** Deposition parameters of thermal barrier coatings.

Spraying Process	Spraying Voltage/V	Spraying Current/A	Powder Feeding Amount/r·min^−1^	Spraying Distance/mm	Spray Gun Rate/mm·s^−1^	Primary Gas (Ar)/KPa	Secondary Gas (He)/KPa
Surface layer	39	850	3.5	85	250	413.7	206.9
Bonding layer	38	750	2.5	85	450	413.7	758.5

**Table 2:** Standard Gibbs Free Energy Comparison Table [17].

Oxidation	Oxidation Process Chemical Formula	Temperature(K)	Gibbs Free Energy (KJ/mol)
Al_2_O_3_	2Al + 1.5O_2_ = Al_2_O_3_	1372	−1239.1
Cr_2_O_3_	2Cr + 1.5O_2_ = Cr_2_O_3_	1372	−769.6
CoO	Co + 0.5O_2_ = CoO	1372	−135.7
NiO	Ni + 0.5O_2_ = NiO	1372	−122.8

**Table 3:** Growth rates of TGO regions under different oxidation processes.

First Stage	KPeak (μm·s^−0.5^)	Kother (μm·s^−0.5^)	Second Stage	KPeak (μm·s^−0.5^)	Kother (μm·s^−0.5^)
Al_2_O_3_	2.755 × 10^−5^	1.895 × 10^−5^	Al_2_O_3_	1.186 × 10^−5^	8.886 × 10^−6^
MO	0	0	MO	6.454 × 10^−5^	3.791 × 10^−5^

**Table 4:** Material parameters of 8YSZ coatings.

Type of Material Physical Parameters	TC	MO	Al_2_O_3_	BC	SUB
Temperature range (°C)	20–1100	20–1100	20–1100	20–1100	20–1100
Elastic modulus (GPa)	48–22	100	400–320	200–110	220–120
Poisson’s ratio	0.1–0.12	0.3	0.23–0.25	0.3–0.33	0.31–0.35
Coefficient of thermal expansion (10^−6^/°C)	9–12.2	5–8	8–9.6	13.6–17.6	11.8–18.7

**Table 5:** Creep effect parameters of 8YSZ coatings.

Material	B (s^−1^MPa^–n^)	n	T (°C)
TC	1.8 × 10^−7^	1	1000
Al_2_O_3_	7.3 × 10^−10^	1	1000
MO	5 × 10^−10^	1	1000
BC	6.54 × 10^−19^	4.57	600
	2.2 × 10^−12^	2.99	700
	1.84 × 10^−7^	1.55	800
	2.15 × 10^−8^	2.45	850
SUB	4.85 × 10^−36^	1	20
	2.25 × 10^−9^	3	1200

**Table 6:** Relationship between number of thermal cycles, TGO growth behavior, stress and cracking.

Simulation Cycles	0	4	6	10	16	20
True Cycles	0	50	200	400	550	750
TGO Equivalent Thickness (μm)	0	0.64	1.64	2.74	5.57	7.94
Growth components	Al_2_O_3_				MO	
Maximum stress σ_xx_(MPa)	−141.86	−47.01	−131.89	+1097.70	+1105.70	+1047.00
Hazardous location	Valley area					
L_V_ (μm)	0	0.52	1.14	3.77	24.45	39.37
Maximum stress σ_yy_(MPa)	+108.13	+116.18	+116.07	+240.72	+252.84	+256.61
Hazardous location	Peak area					
L_P_ (μm)	0	0.64	1.06	4.61	18.76	34.31

## Data Availability

Not applicable.
